# Supplementary figures and images for: Effects of thiourea on the skull of Triturus newts during ontogeny
Source: PeerJ. 2021 Jun 2;9:e11535. doi: 10.7717/peerj.11535 (PMC8179219; doi:10.7717/peerj.11535)

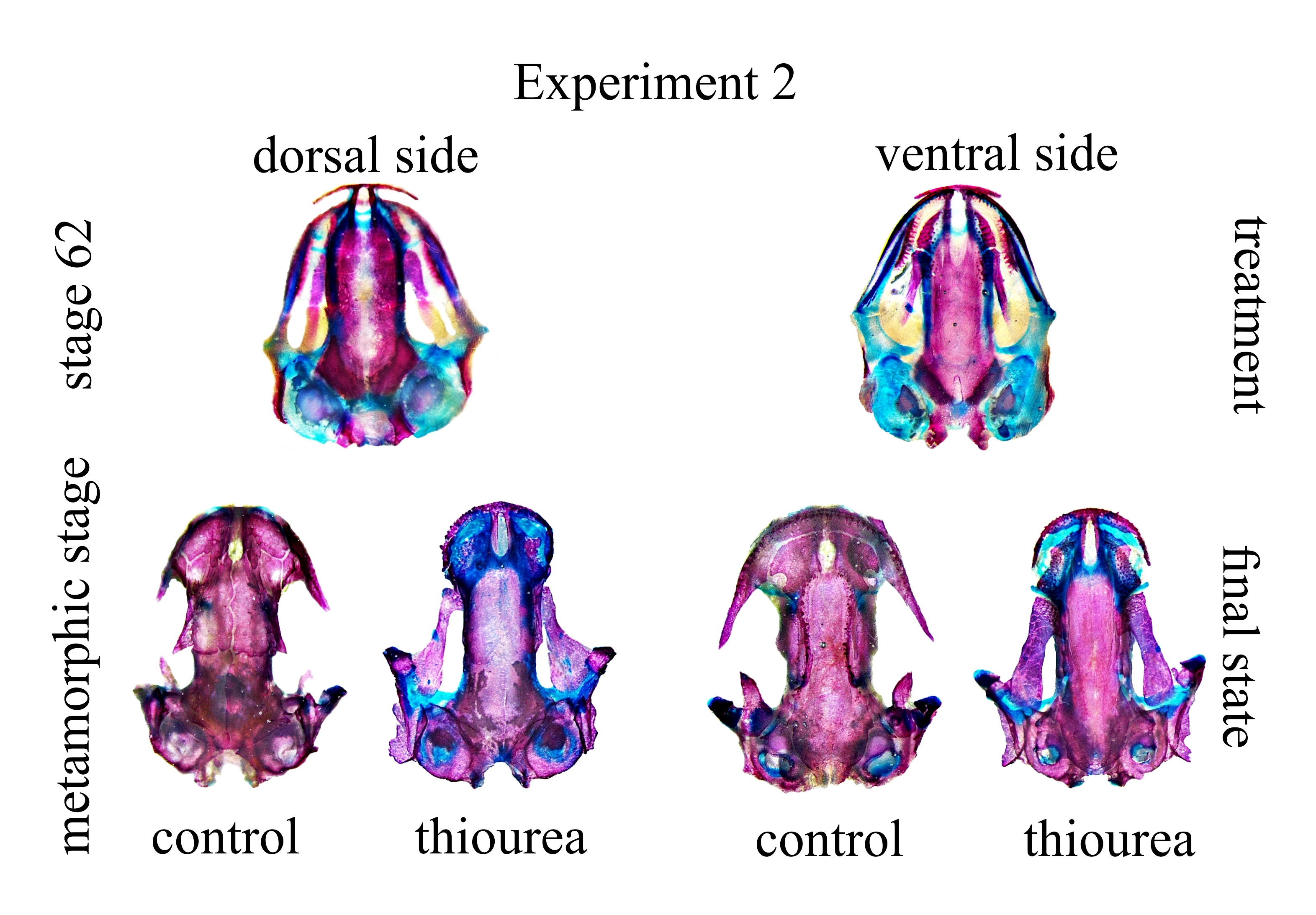

Supplement: Supplemental Information 1 [file peerj-09-11535-s001.jpg]
