# Supplementary material for: Effects of thiourea on the skull of Triturus newts during ontogeny
Source: PeerJ. 2021 Jun 2;9:e11535. doi: 10.7717/peerj.11535 (PMC8179219; doi:10.7717/peerj.11535)
Supplement: Supplemental Information 2 [file peerj-09-11535-s002.docx]

**Table S1.** Graphical presentation of analyzed stages (from hatching until metamorphosis) with brief description of developmental stages

| **Stage** | **Limb differentiation and larval description** |
| --- | --- |
| 42-43 | Hatchlings: beginning of formation of the first two digits in forelimbs. |
| 44 | The first two digits have a cone like shape and both are the same length. |
| 45 | The two front digits have typical toe shape, they are not more cone like. The 2^nd^ digit is slightly longer than the 1^st^ digit. Gills are richly branched and regression of balancers begins. |
| 46 | Beginning of 3^rd^ digit of fore limb appeared. |
| 47-48 | Further elongation of 3^rd^ digit is evident. Gills are long and branched. |
| 49 | The 1^st^ and 3^rd^ digits are the same length, but the 2^nd^ is slightly longer. |
| 50-52 | Beginning of 4^th^ digit of fore limb appeared. Hind limb bud appears. |
| 53 | Elongation of 4^th^ digit of fore limb continues and hind limb bud displays a cylindrical shape. Balancers are completely reduced. |
| 54-55 | Hind limb shows a bifurcation distally. |
| 56 | Hind limb has two digits well formed. |
| 57 | Beginning of 3^rd^ digit of hind limb appeared. |
| 58 | All digits of fore limb are well formed and 3^rd^ digit of hind limb continues to grow. |
| 59 | Hind limb displays a 4^th^ digit bud. |
| 60 | Elongation of all digits of hind limb is evident. |
| 61 | Hind limb displays a five-digit bud. |
| 62-63 | All five digits of hind limb are well formed. Larvae characterized by fully develop limbs. The gills reach their maximum. Skin on the dorsal body side is yellowish colour with rounded melanophores. |
| Metamorphosis | The gills are resorbed and gill slits are completely closed. Skin is darker with white dots spread along the midline of the body |
| 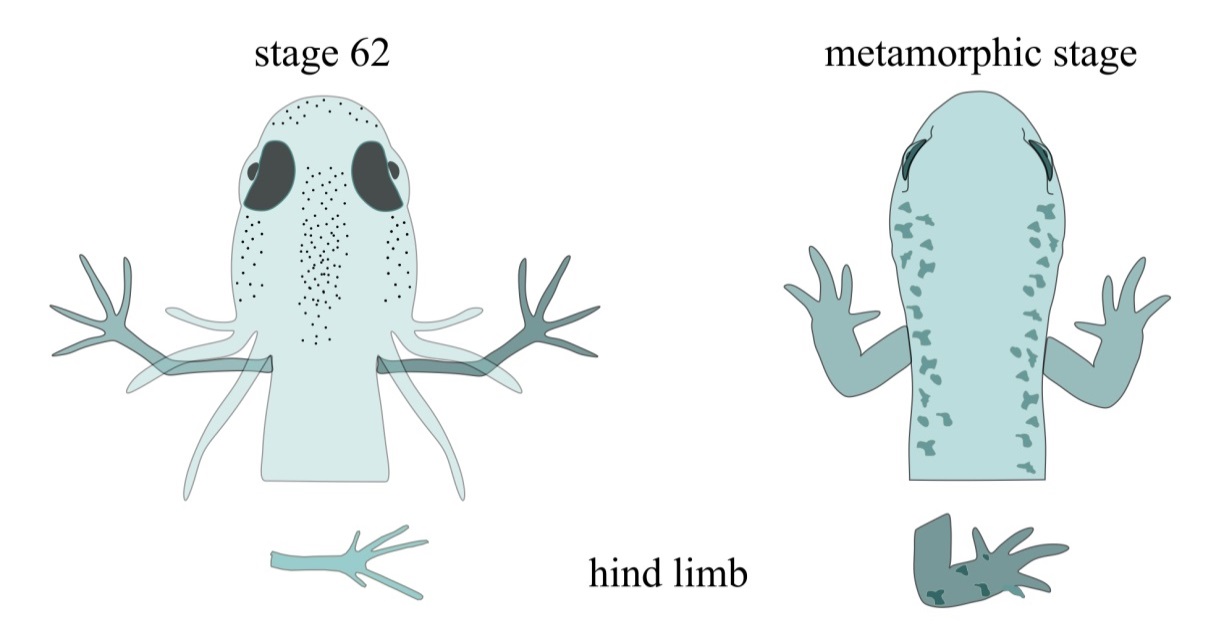 | |
