# Supplementary material for: Effects of thiourea on the skull of Triturus newts during ontogeny
Source: PeerJ. 2021 Jun 2;9:e11535. doi: 10.7717/peerj.11535 (PMC8179219; doi:10.7717/peerj.11535)
Supplement: Supplemental Information 3 [file peerj-09-11535-s003.docx]

**Table S2**

Brief anatomical description of the position of cranium landmarks for two ontogenetic stages.

| Dorsal side | Landmark | Position |
| --- | --- | --- |
| Stage 62 | 1 | Anterior tip of premaxilla |
|  | 2, 3 | Lateral tip of premaxilla |
|  | 4, 5 | Most anterior point of otic capsule |
|  | 6, 7 | Most posterior point of otic capsule |
|  | 8 | Posterior end of parietal bones |
| Metamorphic  stage | 1 | Anterior tip of premaxilla |
|  | 2, 3 | Lateral tip of premaxilla |
|  | 4, 5 | Suture between frontal and parietal |
|  | 6, 7 | Suture between parietal and squamosum |
|  | 8, 9 | Most posterior point of occipital |
|  | 10 | Medial tip of the occipital |
| Ventral side |  |  |
| Stage 62 | 1 | Anterior tip of premaxilla |
|  | 2,3 | Lateral tip of premaxilla |
|  | 4, 5 | Quadrate, most lateral jaw point |
|  | 6, 7 | Lateral tip of occipital condyle |
|  | 8 | Most posterior point of parasphenoid |
| Metamorphic stage | 1 | Anterior tip of premaxilla |
|  | 2, 3 | Lateral tip of premaxilla |
|  | 4, 5 | Quadrate, most lateral jaw point |
|  | 6, 7 | Lateral tip of occipital condyle |
|  | 8 | Most posterior point of parasphenoid |
